# *Toxoplasma gondii* Oocyst–specific Antibodies and Source of Infection

**DOI:** 10.3201/eid1610.091674

**Published:** 2010-10

**Authors:** Claudia A. Muñoz-Zanzi, Paulina Fry, Blaz Lesina, Dolores Hill

**Affiliations:** Author affiliations: University of Minnesota, Minneapolis, Minnesota, USA (C. Muñoz-Zanzi);; Universidad Austral de Chile, Valdivia, Chile (C. Muñoz-Zanzi, P. Fry, B. Lesina);; United States Department of Agriculture, Beltsville, Maryland, USA (D. Hill)

**Keywords:** Toxoplasma gondii, oocysts, infection source, transmission, sporozoite-specific protein, parasites, dispatch

## Abstract

Infection source can determine cost-effective public health interventions. To quantify risk of acquiring *Toxoplasma gondii* from environmental sources versus from meat, we examined serum from pregnant women in Chile. Because 43% had oocyst-specific antibodies, we conclude that contaminated meat remains the primary source of infection but that environmental sources also pose substantial risk.

Toxoplasmosis is a zoonotic disease that occurs worldwide and is caused by the protozoon *Toxoplasma gondii*. The public health relevance of toxoplasmosis relates to congenital (*1*) and postnatal infection (*2*–*4*). The distribution of postnatal infection is highly variable worldwide, or even within a country, probably because of environmental, socioeconomic, and cultural factors. Seroprevalence estimates range from 11% in the United States (*5*) to >70% in Brazil (*6*). Postnatal infection is caused by ingestion of undercooked meat containing tissue cysts; ingestion of water, fruits, vegetables, and shellfish contaminated with oocysts; or unintentional ingestion of cat feces or soil that contain oocysts (*7*,*8*). Population studies to determine specific risk factors for infection and source attribution have been based on the epidemiologic analysis of information from questionnaires administered to infected and uninfected persons; however, applicability of this method is limited. The relative roles of various potential sources are not known and probably vary from population to population. Knowledge of sources of infection within a specific type of community can provide valuable information for designing cost-effective food safety and public health interventions. Our objective, therefore, was to quantify the risk of acquiring infection from environmental sources (oocysts) compared with the risk from eating meat. We did so by detecting antibodies against a recombinant sporozoite-specific protein (SSP), which is found only in the oocyst (sporozoite) stage of the parasite (*9*,*10*).

## The Study

We used 494 banked serum samples from pregnant women in Valdivia Province, southern Chile, who had participated in an unrelated study. *T. gondii* prevalence in this population was high (39%). A convenience sample was selected from among women who were at least 18 years of age at the time of their first prenatal visit at 1 of 5 public clinics, which served mostly low-income persons from urban communities. Information available was age, gestation time, residency (urban or rural), socioeconomic status, and education level. Because of the small sample size, p<0.1 was considered significant. The study protocol (0901E57685) was approved by the University of Minnesota Institutional Review Board.

Past exposure to *T. gondii* was determined qualitatively by immunoglobulin (Ig) G, detected by using a commercial enzyme immunoassay kit (*Toxoplasma* IgG EIA, Bio-Rad Laboratories, Redmond, WA, USA). According to the manufacturer, sensitivity and specificity of this assay were 83% and 93%, respectively. Approximate timing of infection for patients with IgG-positive samples was ascertained by using a commercial solid-phase enzyme immunoassay (*Toxoplasma gondii* IgG Avidity EIA; Ani Labsystems Ltd. Oy, Vantaa, Finland) to determine avidity (binding ability) index. According to manufacturer recommendations, results were interpreted as follows: avidity <15%, acute infection; 15%–30%, possible infection during the past 6 months; and >30%, infection not within the past 3 months.

A Western blot assay developed at the Animal Parasitic Diseases Laboratory of the US Department of Agriculture, with estimated sensitivity and specificity of 88% and 100%, respectively, was used to qualitatively evaluate IgG-positive serum samples for antibodies to SSP. Details of the assay protocol and validation procedures have been submitted for publication (D. Hill et al., unpub. data, www.ars.usda.gov/research/projects/projects.htm?ACCN_NO=409642&showpars=true&fy=2009).

A total of 193 samples were positive for *T. gondii* IgG; overall seroprevalence was 39.1% (90% confidence interval [CI] 34.9%–43.5%) (Figure). Age of seropositive women was higher than that of seronegative women (p = 0.011). In addition, college-level education was protective for *T. gondii* exposure (p = 0.077) (Table). Of 180 *T. gondii* IgG–positive women with available SSP results, 64 (35.5%) had SSP antibodies. Validation of the SSP assay indicated that SSP antibodies tend to decrease over time and can become undetectable after 6–8 months (Hill et al., unpub. data); therefore, we calculated the proportion of the study population with SSP antibodies among women with evidence of recent (within the past 6 months) infection only. Of the 193 *T. gondii*–seropositive women, 72 (32.6%) had evidence of recent or acute infection (avidity <30%; Figure), although this was probably an overestimation because avidity increases slowly in certain persons. Among these 72 women, 31 (43.1%) had SSP antibodies, including 4 (45.8%) of 9 acutely infected women and 27 (44.4%) of 59 women with evidence of recent infection (Figure). Socioeconomic status was the only demographic factor significantly associated with SSP antibodies (p = 0.056). The age-adjusted odds ratio for socioeconomic status was 4.06 (90% CI 1.21–13.60), indicating that the odds of having SSP antibodies was 4× higher for women of very low (<$3,500/year) compared with low (>$3,500/year to <$5,000/year) socioeconomic status.

**Figure:**
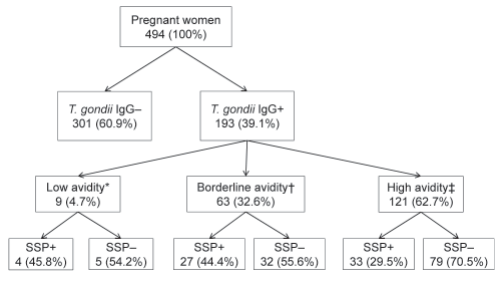
Detection of *Toxoplasma gondii* in 494 low-income pregnant women from Valdivia Province, Chile. *<15%, indicates acute infection; †15%–30%, indicates possible infection within <6 mo (4 samples from recently infected women were not tested for SSP antibodies); ‡>30%, excludes recent (within 3 mo) infection (9 samples from women with chronic infection were not tested for SSP antibodies). –, negative; +, positive; Ig, immunoglobulin; SSP, sporozoite-specific protein antibodies.

**Table:** Demographics of low-income pregnant women evaluated for exposure to *Toxoplasma gondii*, Valdivia Province, Chile*

Variable	Seronegative	Seropositive	Total	Odds ratio (p value)†
Urban residence‡	289/300 (96.3)	184/189 (97.4)	473/489 (96.7)	– (0.538)
Very low socioeconomic status§	261/300 (87.0)	167/191 (87.4)	428/491 (87.2)	– (0.888)
College/technical education¶	41/300 (13.7)	16/191 (8.4)	57/491 (11.6)	0.58 (0.077)
Age, y, median#	25 (18.0–39.0)	27 (18.0–41.0)	25 (18.0–40.2)	1.46** (0.011)
Total, no. (%)	301 (60.9)	193 (39.1)	494	

*Values are no./no. tested (%) except as indicated. †Odds ratio and corresponding p value from univariate logistic regression analysis. ‡Reference is rural residence. §Household income <$3,500/y; reference is low socioeconomic status, household income >$3,500/y to <$5,000/y. ¶Reference is no college or technical education. #Median, 2.5th, and 97.5th percentiles (in parenthesis) are reported for age as a continuous variable. **Odds ratio for a 28-year-old woman compared with an 18-year-old woman as an example.

## Conclusions

Our finding that 43% of toxoplasmosis infections were associated with direct or indirect exposure to an occyst-contaminated environment indicates that cyst-containing meat was the major (57%) source of infection in the study population (Figure). Although this value is likely to vary by population, it is consistent with findings from a multicenter case–control study that reported, on the basis of calculation of population-attributable fractions, that 30%–63% of acute infections could be attributed to eating meat (*11*). Although data on prevalence of infection in meat-producing animals from the area are limited, in general, the high risk for human infection is consistent with the high seroprevalence reported for swine (9%) (C.A. Muñoz-Zanzi et al., unpub. data), free-range chickens (55%) (*12*), and sheep (28%) (*13*). In communities such as our study area, consumption of meat produced locally from small farms and backyard pens is common, especially for populations of low socioeconomic status. In these informal farm management systems, animals are at higher risk of acquiring *T. gondii*.

Our detection of SSP antibodies in 43% of recently infected women (Figure) implies that infection from ingestion of oocysts is an almost equally large public health problem in this population as is contaminated meat. A high level of oocyst contamination of soil and especially water (because of runoff) probably results from high (33%) prevalence of infection in cats (*14*) and high annual precipitation. Oocysts in the local environment have not been studied; however, infection of local aquatic or semiaquatic species, such as sea lions and feral mink (M. Sepulveda, unpub. data), suggests that surface water is contaminated with oocysts. Although less recognized than contact with cat feces (while handling cat litter), eating raw shellfish, which can accumulate oocysts (*7*), and drinking contaminated water are major risk factors for *T. gondii* infection (*15*). Use of a study population of pregnant women affects generalizability of findings; nevertheless, results indicate that toxoplasmosis is a major public health problem in this area of Chile. Despite knowledge of how to prevent or minimize risk for infection (e.g., cook meat thoroughly, freeze meat, wash vegetables, wash hands after handling raw meat and after gardening), the high risk for infection in this population highlights the need for improved education programs, especially for populations of low socioeconomic status.

A study limitation is the possibility of misclassifications (false-positive or false-negative results) with use of the IgG, SSP, and avidity assays. Despite this limitation, the fraction of infection attributed to oocysts was consistent for all infected groups. A method for quantifying error rates, sensitivity, and specificity of the entire testing algorithm is being developed. Lastly, the potential effects of strain variability of immune response and detection of SSP antibodies remain unknown for this new tool.

The new tool reported here can be used for source attribution as well as epidemiologic analysis of self-reported data. Its use should lead to improved effectiveness of intervention programs.
